# Prelamin accumulation in primary endothelial cells induces premature senescence and activation

**DOI:** 10.1186/1750-1172-10-S2-O5

**Published:** 2015-11-11

**Authors:** Nathalie Bonello-Palot, Catherine Badens

**Affiliations:** 1 Aix Marseille Université, INSERM, GMGF UMR_S 910 & Department of Medical Genetics and Cell Biology, La Timone Children's hospital, APHM, 13385, Marseille, France

## 

Defects in lamin A maturation result in premature aging syndromes and severe atherosclerosis as observed in Hutchinson-Gilford Progeria Syndrome. In age-related atherosclerosis, several features of cells senescence have been characterized in endothelial cells lamin A alterations. We propose a cellular model to study lamin A-related senescence in primary endothelial cells. In this model, lamin A defects were induced by protease inhibitor (PI: Atazanavir) treatment during 48h on normal cells issued from placenta (human umbilical vein (HUVEC) or cord blood (ECFC)). We showed that PI treatment led to the accumulation of farnesylated prelamin A and induced nuclear shape abnormalities and premature senescence in both HUVEC and ECFC. ICAM-1-dependent activation was present and monocytes adhesion was increased in HUVEC whereas ability to generate microvascular network in matrigel was decreased for ECFC. The effects of PI treatment on nuclei shape were reversed when cells were PI-treated in combination with Pravastatin and Zoledronate in both mature and progenitor endothelial cells. Reversion also was demonstrated with 2 antisens-oligonucleotides targeted toward lamin A specific splice sites. This study confirms that PI treatment reproduces premature senescence due to lamin A maturation defects in primary endothelial cells after a 2 days exposure. The cells used were extracted from full term and healthy neonates i.e. from individuals of age 0. This allows us to consider that other senescence pathways were not activated and that the observed alterations were specific of prelamin A accumulation. This model constitutes a valuable tool to test different approaches aimed at reversing specifically lamin A-related cells senescence.

